# Coping strategies adopted by caregivers of children with autism in the Limpopo Province, South Africa

**DOI:** 10.4102/ajod.v13i0.1384

**Published:** 2024-09-30

**Authors:** Gsakani O. Sumbane

**Affiliations:** 1 School of Medicine, Faculty of Health Sciences, University of Limpopo, Polokwane, South Africa

**Keywords:** Autism spectrum disorders, caregivers, emotion-focused coping, problem-focused coping, strategies

## Abstract

**Background:**

Emotion- and problem-focused coping strategies are frequently employed by caregivers of autistic children to increase their general well-being and resilience to the stress of raising the child. Although these strategies cannot directly address the situation, it is useful for handling stressful situations that cannot change.

**Objectives:**

The study seeks to explore and understand the emotion- and problem-focused strategies that caregivers use to cope with the challenges of raising a child with autism.

**Method:**

This was a qualitative, exploratory and descriptive research study. Twenty-eight caregivers were purposive-convenient sampled from the public special schools where their autism spectrum disorder (ASD) children are schooling. Semi-structured interviews were used to gather data, which were then transcribed and subjected to thematic analysis.

**Results:**

Positive emotion-focused strategies include positive reappraisal, reframing and acceptance. Negative emotion-focused strategies include denial, emotional release, cognitive distortion, self-isolation, overprotection, negative self-talk, punishment and religion. Problem-focused coping strategies include active and adaptive coping, peer group, professional support and social support.

**Conclusion:**

The coping methods that have been identified can be integrated into intervention programmes and serve as a guide for specialised institutions that offer more extensive knowledge and assistance to families who are caring for children with ASD.

**Contribution:**

The study contributes to understanding the emotion- and problem-focused strategies adopted by rural caregivers in managing their emotions, interpretation of the situation and adjusting to the demands of raising an autistic child.

## Introduction

Caregivers of children with autism spectrum disorder (ASD) experienced elevated levels of stress and frequent physical and mental burnout (Harris 2021). The ASD literature shows that caregivers’ stress is influenced by the child’s behavioural problems and deficits in socialisation and communication (Amireh 2019). Caregivers of children with autism take on excessive responsibilities such as meeting social, physical, emotional and learning needs of a child (Marsack-Topolewski & Wilson 2021), and this situation causes stress, despair and anxiety (Ishtiaq, Mumtaz & Saqulain 2020). To deal with stress, caregivers of children with ASD use coping strategies that help to tackle the challenging situations of raising the child (Ismail Mohd et al. 2022; Megreya et al. 2020). Two dimensions of coping strategies mostly used include problem-focused coping, and the purpose is to solve the problem or take action to change the status quo (Ghanouni & Hood 2021). The other one is an emotion-focused coping, which aims to reduce the emotional distress associated with stressful situations (Vernhet et al. 2019).

The literature showed that female caregivers, like mothers of the ASD children, used emotion-focused coping more than fathers (Haytham et al. 2022). The most commonly used strategies include crying, spiritual behaviours and acceptance, which are reported to be the most crucial elements of female caregivers coping strategies (Balubaid & Sahab 2017; Wei-Chih, Chang & Kuo-Yu 2023). However, the emotion-focused strategy such as denial, avoidance and social withdrawal have generally been found to be associated with higher levels of psychological distress on the caregivers (Manicacci et al. 2019; Salimi et al. 2019). Using problem-focused coping strategies, such as seeking information, seeking social support, seeking professional help and taking action to address the problem, has been linked to better mental health outcomes (Harris 2021; Ishtiaq et al. 2020). According to Haytham et al. (2022), positive coping strategies increase the likelihood of smoother interactions between ASD children and their caregivers, as well as increased acceptance and less rejection from society.

Caregivers of children with ASD, however, differ in how they cope with perceived stressors based on a variety of factors (Gagat-Matuła 2022; Vanmeter, Handley & Cicchetti 2020), including the circumstance or threat being faced, personality traits, positive beliefs, culture, social support networks, problem-solving skills and religion (Pisula & Banasiak 2020; Yaacob et al. 2022). On the other hand, it has also been proposed that having an ASD child may impact the quality of life for the caregiver in addition to being associated with emotional stress and several social, physical and financial factors (Wei-Chih et al. 2023).

**FIGURE 1:**
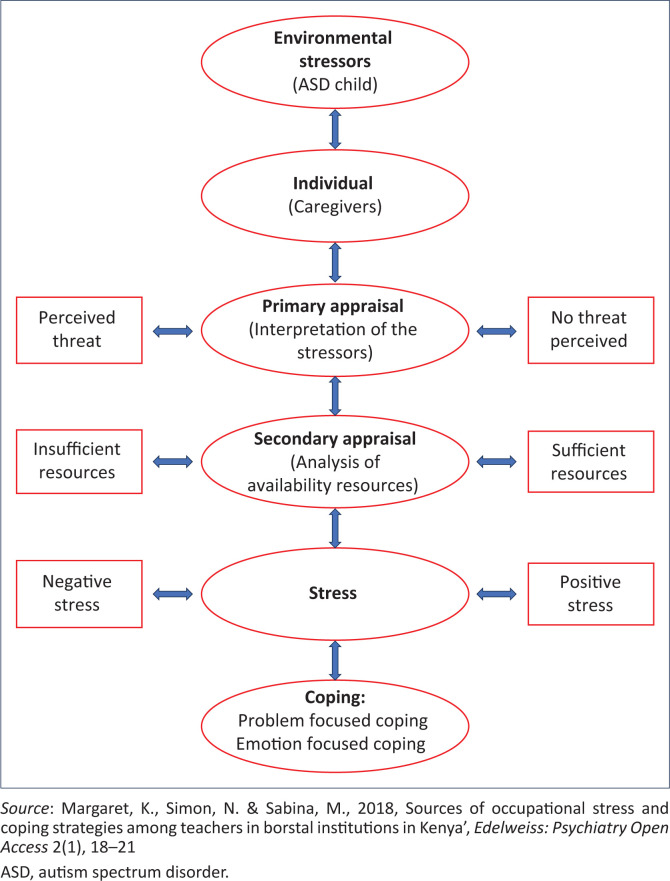
Transactional model of stress and coping.

The study was guided by Lazarus and Folkman’s Transactional Model of Stress and Coping. The framework illustrates how major life events affect human emotions and help people cope with stressful situations by using cognitive appraisal and coping strategies (Obbarius et al. 2021). The focus of the theory lies on cognitive assessment and dealing with stress and coping. The three major concepts of the model include stress, appraisal and coping (Lim et al. 2023) as illustrated in Figure 1. According to the transactional model, people experience stress when the demands of a situation exceed their capacity to deal with it (Furrukh & Anjum 2020). The amount of stress people experience is based on their assessment of two factors: the assessment of the stressors (called primary appraisal) and the assessment of the resources they must cope with (called secondary appraisal) (Bhattacharya et al. 2019). The model has identified two general coping strategies (Loewenstein, Barroso & Phillips 2019): one is a problem-focused coping, the purpose is to solve the problem or take action to change the status quo; the other is an emotion-focused coping, which aims to reduce the emotional distress associated with stressful situations. When effective coping strategies are adopted, the individual will overcome, adjust and learn from the stressful situation (Lim et al. 2023). However, the ineffective coping strategies will affect the adjustment to the situation and the situation will be perceived as a threat or dangerous (Rosli et al. 2020). Caregivers of children with ASD are faced with a major life event of raising a child with ASD which might affect their emotions or adjustment. The framework will guide on the assessment of the coping strategies adopted in managing the emotional distress associated with raising a child with ASD. Figure 1 summaries the main concepts of the theory.

To date, most of the studies that have targeted caregivers of children with ASD were conducted in western or developed eastern countries (Reddy, Fewster & Gurayah 2019). No studies have investigated the coping strategies of caregivers providing care to children with ASD in the Limpopo province. Different cultural ideas held by caregivers in the Limpopo province have influenced how they interpret ASD and make decisions (Numisi et al. 2020). It is noted in the article written by Mojela (2020) and Sumbane et al. (2023) that the lack of resources associated with ASD, a higher level of stigma in society and inadequate knowledge could result in psychosocial stress to parents and caregivers. The lack of local literature and data about coping strategies makes this study essential. It might serve as a strong foundation for upcoming studies.

## Research methods and design

### Research design

An exploratory and descriptive research methodology was employed to better understand how caregivers of ASD children coped with the stressful aspects of their child by collecting data directly from the caregivers themselves. Therefore, the researcher described participants’ spoken and written words and interpretation of observed behaviour in a way the participants explained them during data collection.

### Setting

The study was conducted in eight public special school in the deep rural areas of the Limpopo province. Special schools are separate schools or classes, specifically designed for students categorised as having special educational needs. The three schools were selected from the Capricorn district, two from the Waterberg and Vhembe districts, respectively, and one each from the Sekhukhune and Mopani districts.

Children in these schools require additional support and adaptive pedagogical methods to participate and meet learning objectives in an educational programme. The selected schools admit around 288 to 380 learners with different physical, behavioural, intellectual, emotional and social capacities. The schools were chosen because they had the highest number of learners with ASD in the Limpopo province. The number of children with ASD from these school were between 6 and 23. Only caregivers of children with ASD participated in the study.

### Sampling of participants

Participants were recruited though the special schools where their ASD children are admitted. Appointments were made with the school principals whereby the researcher presented her study by explaining what the study entailed and its purpose. The school principals arranged a meeting with the school governing bodies and caregivers for the researcher to inform them about the study and explain its purpose. English or Xitsonga, Sepedi and Tshivenda information sheets were provided to the caregivers together with consent forms. Those who were willing to participate were requested to sign the consent form. For those who were unable to attend the meeting, their ASD children were handed the informed consent form and information sheet to present to their caregivers, asking them to indicate whether they would like to participate or not and returning the form to the school with the child.

Twenty-eight caregivers were selected through the purposive-convenient sampling method. The participants consisted of eighteen mothers, two grandmothers, one uncle, one aunt, one helper, one brother and five fathers. The selected participants had experience in caregiving for children with ASD, were staying with the child in the same household and engaged in the child’s day-to-day care. Eighteen participants were mothers of children with ASD, followed by five fathers, two grandparents and one aunt, brother and uncle, respectively. Most of the participants were of middle age, and all grandparents were pensioners above 60 years. Majority rely on small-scale farming, social grants and lowest paying jobs for survival. Their ASD children were between 7 and 17 years of age of which most of them were boys. Table 1 summaries the participants’ characteristics.

**TABLE 1:** Participants characteristics.

Participant number	Marital status	Age	Location or district	Employment status	Role	Age of the ASD child
1	Married	50	Capricorn	Employed	Mother	17
2	Single	27	Capricorn	Employed	Brother	9
3	Widow	64	Capricorn	Pensioner	Grandmother	16
4	Married	65	Capricorn	Pensioner	Grandmother	15
5	Married	35	Capricorn	Employed	Mother	9
6	Single	47	Capricorn	Unemployed	Mother	17
7	Single	30	Capricorn	Unemployed	Mother	10
8	Single	29	Capricorn	Employed	Mother	10
9	Married	48	Waterberg	Employed	Mother	17
10	Widow	46	Waterberg	Unemployed	Mother	9
11	Single	25	Waterberg	Unemployed	Mother	7
12	Single	40	Waterberg	Unemployed	Mother	10
13	Single	35	Waterberg	Employed	Mother	17
14	Single	43	Waterberg	Unemployed	Aunt	16
15	Single	27	Sekhukhune	Unemployed	Mother	7
16	Single	49	Sekhukhune	Unemployed	Mother	12
17	Single	44	Sekhukhune	Employed	Helper	10
18	Single	31	Vhembe	Employed	Mother	10
19	Single	32	Vhembe	Employed	Mother	10
20	Married	36	Vhembe	Unemployed	Mother	9
21	Married	54	Vhembe	Employed	Father	14
22	Married	51	Vhembe	Employed	Father	14
23	Married	53	Vhembe	Employed	Father	17
24	Single	37	Vhembe	Employed	Mother	10
25	Married	51	Vhembe	Unemployed	Mother	11
26	Married	43	Vhembe	Employed	Father	7
27	Married	49	Mopani	Employed	Father	13
28	Married	43	Mopani	Employed	Mother	10

ASD, autism spectrum disorder.

### Data collection

Data were collected by the researcher at the venue that was convenient for the participant either at the selected special school during school hours in a private room or at their homes. The selected venue was spacious and comfortable for participants. Face-to-face semi-structured interviews were chosen to address this study’s research questions and objectives. To explore the participants’ experiences, the researcher started with a general question, ‘can you share with me the strategies that you use to cope with raising a child with autism’. The researcher used probing to understand better how this phenomenon was experienced. The probing question used was: ‘Regarding the situations that were particularly stressful what steps did you take then?’ A notebook and pen were used to record and keep field notes during the observations and watch verbal and non-verbal communication. A tape-recording device audio-recorded the conversation during individual interviews. The researcher sought the participant’s permission to take notes and record the interviews. Data were collected until data saturation. After the 20th interview, the data became saturated; however, eight more interviews were conducted by the researcher to validate the saturation of the data.

### Data analysis

The initial step in data analysis was to transcribe the unprocessed interview data. After every interview was finished, audio-recorded interviews were transcribed verbatim.

In this study, thematic analysis was utilised. To get a sense of the caregivers’ viewpoints and overall meaning, the researcher and the supervisor first critically reread the first five transcripts (Creswell 2014). After that, we coded the transcripts, and the researcher produced an initial list of nodes in the NVivo^®^. Prior to evaluating the themes to create an overall group analysis, we individually examined each transcript. Then, we arranged the themes into interconnected hierarchies (i.e., themes, subthemes and categories).

After that, a meeting is scheduled to decide on the codes. This triangulation procedure produced the necessary codes that the researcher could use to continue the data analysis process, hence reducing the inter-rater difference.

To enhance trustworthiness and verify coding precision, the research supervisors examined the initial units of the meaning code for every interview transcript. They discussed any discrepancies after comparing the data analysis and themes to a consistency check. Furthermore, following a review of the coding units for five transcripts and the themes or subjects for the entire sample, the co-supervisor, a skilled qualitative researcher, offered general remarks and recommendations. Once the data analysis was finished, the research team met once more to decide on the final codes as well as the themes, subthemes and categories.

The co-corder validated and confirmed the themes by reading and evaluating each transcript, looking over the themes, supporting information and transcripts again. The theme was excluded if the co-corder and the researcher discovered data or information that did not support the themes or the experiences of the caregivers. After reaching a consensus, the ultimate themes were determined.

### Ethical considerations

Ethical clearance to conduct this study was obtained from the University of Limpopo, Turfloop Research Ethics Committee (No. TREC/232/2016:PG). The Department of Basic Education and the special school principals granted permission for the data collection.

The researcher first visited the selected special schools to request permission from the principals of the schools and to explain the outline of the study and its purpose. The school principals organised the caregivers on a set date for the researcher to inform them regarding the study. The researcher outlined the purpose, objectives and importance of the study and invited them to participate in the study. The date and time for the interviews were set for those who gave consent. Prior to the interviews, study participants signed an informed consent form, indicating that their participation was voluntary. The researcher had no relationship with the study setting.

## Results

Two themes and fourteen subthemes emerged from the results as illustrated on Table 2. The study showed that the caregivers use strategies that were mostly likely to be positive and negative to cope with the caring and raising of a child with ASD. Below is the description of themes and subthemes:

**TABLE 2:** Study findings.

Themes	Subthemes
Negative emotion-focused coping strategies	Denial Emotional release Cognitive distortions Overprotection Self-isolation Child isolation Negative self-talk Corporal punishment
Positive emotion-focused coping strategies	Reframe Acceptance Re-interpretation or re-appraisal Religious believes
Problem-focused coping strategies	Adaptive coping Active coping Peer-group emotional support Professional emotional support Social emotional support

### Theme 1: Negative emotion-focused coping strategies

The negative emotion-focused coping strategies according to this study were those strategies that negatively influence the caregiver’s adaptation to the situation. Some of these strategies encourage them to refuse to accept the reality, view the situation as a threat, isolate themselves from others and release their emotions as discussed below.

#### Denial

The caregivers expressed that when they first learned that their children had autism, they were upset and doubted themselves. Several caregivers admitted that they initially refused to accept the fact that their children had autism. They expressed that they had frequently questioned why they have this child. While others expressed to have spent sleepless nights, others used to lock themselves in their homes. They felt this way because none of their families had a child with autism; some even claimed to be in denial because they were unaware of the condition. As evidenced by:

‘At the beginning I could not believe it, I use to wake up at night and ask myself why God gave me this child, I could not accept him. I used to lock myself in the house and ask myself too many questions because number one I did not even understand what autism is.’ (P3, Widow, 64 years old, Capricorn)

‘I used to ask myself “why me” why am I having a child like this one, because we do not have a child who is like this in my family, I could not believe it.’ (P1, Married, 50 years old, Capricorn)

#### Emotional release

The study found some caregivers, specifically mothers of children with ASD, reported to cope with the stress of raising a child with ASD by expressing their emotions through releasing emotions such as crying. Some caregivers highlighted that they cried because they cannot cope with the burden of caring a child who needs special attention, supervision and strict monitoring.

Caregivers reported that crying helps them feel better, reduces stress and keeps them calm.

Three participants expressed how crying relieves their stress, as evidenced by the following quotations:

‘Crying also helps a lot it makes you become calm and digest things well.’ (P5, Married, 35 years old, Capricorn)

‘Whenever [*I*] am stressed I just keep it to myself I do not like sharing my problems with anyone. Sometimes I would just cry alone until I become fine.’ (P10, Widow, 46 years old, Waterberg)

‘I just go to my room and lock [*the door*] then I would cry to feel better once [*I*] am fine then I can be able to talk.’ (P8, Single, 29 years old, Capricorn)

#### Cognitive distortions

Autism spectrum disorder children tend to do best when they have a highly structured schedule or routine; these include regular times for meals, therapy, school and bedtime. In this study, some of the caregivers highlighted that therapy and school are ineffective for their children. They expressed that they have been taking their children for therapy or school; however, there is no progress. As a result, some caregivers are thinking of switching schools, while others gave up on the therapy. This coping strategy makes them more stressed and makes it more challenging to resolve their situation.

A female caregiver of a 7-year-old boy expressed how she gave up on the therapy, because she thought that it was not working:

‘Since we started taking him for speech therapy and physiotherapy when he was five years old, I did not notice any progress, they didn’t help him with anything, we ended up giving up everything thinking that nothing was working.’ (P17, Single, 44 years old, Sekhukhune)

A male caregiver of a 9-year-old boy expressed that the special school is making his son’s condition worse rather than better, and the situation makes the family to be more stressed:

‘We think that the school is not working for our child, when we took him home during school holidays his progress is slowing down rather than improving. When he is at school, he is shockingly quiet and appears to be lost. We are frustrated as a family because he is not improving, but if he returns home, you will find him engaged and he improves steadily. Even though we have not decided yet, we even thought about changing his school.’ (P5, Married, 35 years old, Capricorn)

#### Overprotection

Seven caregivers expressed that they have an intense fear of being separated from their ASD children. However, they coped very well when they are with their children every day. They made it clear that they did not want their ASD children to attend boarding school. They also feel uncomfortable leaving their children with others if they needed to go somewhere, because other people would not understand their children’s behavioural problems and how to manage it. Instead, they preferred to bring their children everywhere they went. As evidenced by:

‘We do not want her to stay in hostel, we want to take care of her ourselves. We want her to go to school and come back every day, so that even when she is not feeling well, we will be able to see her and take her to the doctor.’ (P16, Single, 49 years old, Sekhukhune)

‘I don’t like to leave him with other people because they won’t understand his needs, like the type of food that he eats.’ (P4, Married, 65 years old, Capricorn)

#### Self-isolation

The caregivers emphasised that because ASD children require continuous care, they are forced to live alone and have no opportunity to see friends or family. A few mentioned that their children are hyperactive and enjoy touching, pressing and damaging objects, which makes them uncomfortable when they visit other people. Some were concerned that their children may become irritated and uncontrollable when they visited others because of unfamiliar surroundings. Additionally, caregivers noted that because of the caregiving responsibilities, it is extremely difficult for them to attend funerals or other family gatherings. Others choose not to attend family gatherings because they worry that the children with ASD may be mistreated there. A few mentioned that they would need to return as soon as possible if they happened to go anywhere. Therefore, caregivers are under pressure because they have to live in isolation to cope with the continual care that children with ASD demand.

An 8-year-old boy’s mother described how she coped with the situation:

‘I cannot visit my friends or anyone because when we arrived there he will touch and breaks other people things. If I left him at home with his aunt, I made sure that I come back as soon as possible, and I told them that they must guard him so that he does not destroy a lot of things while I am away.’ (P14, Single, 43 years old, Waterberg)

An 8-year-old boy’s mother expressed that she uses self-isolation because the community does not treat his son well because of his behavioural problems:

‘It affects me because I do not even attend to family social functions anymore, because of the way they are treating him, and there is no way that I will leave him with other people because of his behaviour. I am used to it; I do not go anywhere.’ (P20, Married, 36 years old, Vhembe)

#### Child isolation

Some caregivers have discovered that the best way to manage raising a child with ASD is to keep the children locked away to prevent them from injuring themselves or others because of their behavioural issues. The caregivers emphasised that they are forced to employ this approach, even though it is not the ideal approach. The mother of a 10-year-old boy who attends a boarding school explained her need to keep the boy hidden during school holidays:

‘I am compelled to put him under lock and key as everyone is terrified of him. He is not allowed to play with other kids. We are not welcomed where we live, which makes the situation challenging. People keep their kids away from him because he used to beat them, but things are much better today. Three of the tenants in our rental rooms left last month because they were terrified of him at home.’ (P9, Married, 48 years old, Waterberg)

Another mother of a 17-year-old girl expressed her situation that forced her to keep the child under lock and key:

‘I do not allow her to get out of the yard because her behaviour is unacceptable and our neighbours, they do not like her. She will open our neighbours’ refrigerator and consume whatever is inside while she is there. That is why I bought a fence so that I will always lock the gate for her. She watches other children playing over the fence and she feels happy by seeing them running up and down.’ (P1, Married, 50 years old, Capricorn)

A mother of a 12-year-old boy expressed how she deals with the situation of taking her child for a follow up at the hospital:

‘When I took her for [*a*] follow up at the hospital, I hire a private transport as she will touch everyone in the taxi and other people, they don’t like it. Some other people will hurt you with their words, which is the reason I am using this strategy.’ (P16, Single, 49 years old, Sekhukhune)

#### Negative self-talk

The study found that the caregivers sometimes experienced an endless stream of unspoken thoughts that ran through their heads. The most negative self-talk was about who will raise their children if they pass away. Caregivers of children with ASD also had ongoing concerns about what would happen to them during puberty or the teenage years because of their low IQs and lack of understanding of what is truly happening. Caregivers of girls with ASD voiced concern that their children might be abused and end up pregnant. Some caregivers were always concerned that having a child with ASD and death are the same thing because it hurts so much.

Most of the caregivers expressed their negative self-talk that really affects their coping skills; one participant who is a mother of a 17-year-old child with ASD highlighted that:

‘Having a child with ASD is just like death is painful’. (P13, Single, 35 years old, Waterberg)

Another participant who was also a mother said that:

‘What will happen to our children when we die, because if we die, they will experience a big problem on their own? (P9, Married, 48 years old, Waterberg)

A 37 old participant who was also a mother of an 8-year gild with ASD expressed the following:

“I have got a fear because my child is a girl, I am just afraid of her puberty and adolescent stage as I don’t know what will happen to her as she doesn’t understand the consequences of sex”.’ (P6, Single, 47 years old, Capricorn)

#### Corporal punishment

Few caregivers said it was challenging to control their children’s behavioural issues; as a result, they end up disciplining them:

‘One day I decided to follow him next door and discipline him, I beat him and then he said, “I am sorry mama”. Since that day he has never behave[*d*] the way, he uses to behaved, I taught him a lesson’. (P8, Single, 29 years old, Capricorn)

‘Is not did you know that children with autism 90% of them are boys sometimes they experience seizures, I used to hit my child during seizures because it was childish behaviour, not realizing that it was an epileptic seizure. I thought that he is just throwing himself down for attention seeking. After the seizure I will scold on the child. However, I felt bad after I was told that it is an epileptic seizure.’ (P3, Widow, 64 years old, Capricorn)

### Theme 2: Positive emotion-focused coping strategies

The results of the study demonstrated that some participants perceived their circumstance as a learning opportunity and analysed it constructively. This allowed them to employ coping mechanisms that lessen the stress associated with raising an autistic child. The following describes the positive emotion-focused coping mechanisms that the caregivers use.

#### Reframe

The caregivers further explained that after dealing with their denial stage, as time went on, they later adopted a more positive outlook on the situation. They emphasised how having a child with ASD had been a benefit for them, as it had allowed them to gain insights and understanding from the circumstance. Mothers of children with ASD who were also teachers at the special school were given the first preference for receiving autism training at work, which benefited their dual roles. The caregivers used the information and experience they had gained from their ASD children to help other parents of ASD children in their communities.

A mother of a 17-year-old autistic child expressed how she chose to see the bright side of the situation and how she helps other women in her neighbourhood who are in similar circumstances. She considered this to be a blessing:

‘At first, it was extremely painful, and I was upset that my child has autism. However, I have since realized that I am fortunate that my child has taught me something new and that I am experiencing something that others are not. Although my knowledge of autism is still limited, however, I will be able to counsel others in need of guidance regarding autistic children. I am glad that because I have a child with autism, the school used to take me to more workshops, specifically on autism. I discovered that my son was doing exactly what they were teaching at the workshops. If they can say, “Write a book about your child”, I will, as it is something I go through every day. I was invited to visit the clinic one day so that I could speak with the mothers of autistic children.’ (P28, Married, 43 years old, Mopani)

Another mother of a 14-year-old autistic child mentioned how his son, who solely speaks English that he has acquired from television, has helped her get better at the language:

‘Although my child could not speak, then I would put him in the TV room and turn on the cartoon channel. It was now that he trained himself to speak English. As for his language, it is solely English. Because I had to speak English with him, my ability to speak in English has therefore improved. Given that some of my family does not speak English, I even translate for them.’ (P21, Married, 54 years old, Vhembe)

#### Acceptance

The study also found that loving and accepting the ASD children was the common strategy that was utilised by the caregivers to cope with the stress of having and raising a child with ASD. Caregivers expressed that even though it takes time to love and accept the child, this strategy is reported to be one of the most important strategies:

‘It took time for me to accept her I started accepting her when she started grade R at the special school when another teacher set me down and explained to me and I started accepting her. It affected me but now I think am fine because whenever I do not see her, I get worried I just want her close to me. I think the first thing that other parents can do is to accept, if you accept your child the way they are it is a start and know that it is not something that will change.’ (P11, Single, 25 years old, Waterberg)

‘The first thing in coping is to love your child; I love him a lot that’s why I can cope.’ (P4, Married, 65 years old, Capricorn)

‘The first thing to cope with is to accept your child. I have accepted my child and even here at home they call him mommy’s child. If he can be out and I do not see him playing outside I get worried.’ (P23, Married, 53 years old, Vhembe)

However, there are caregivers who expressed that it is not easy to accept the situation:

‘[*I*]t is so painful when I start talking about her, even if is its long that she has been there, but the situation is not simple to accept, is just like death is painful.’ (P24, Single, 37 years old, Vhembe)

#### Positive re-interpretation or re-appraisal

The study also found that other caregivers cope by using various positive perspectives to reaffirm or instil hope in their children’s situations such as complementing the children when they help with the house chores, allowing their children interact with other autistic people, motivating ASD children by those who have succeeded in life, perceiving the situation positively despite the negative public attitude and taking the ASD child for an outing to celebrate their birthdays. All these strategies were reported to instil hope in their situation:

‘I always praise her whenever she assists with house chores.’ (P27, Married, 48 years old, Mopani)

’When coming to house chores he doesn’t have a problem because he helps a lot.’ (P13, Single, 35 years old, Waterberg)

‘We even tried taking her to relatives in Polokwane where they have children with autism so that she can learn to accept herself and that there are people like her because she used to believe she is the only one like that. Since she came to this school even when people call her by names she does not get bothered.’ (P7, Single, 30 years old, Capricorn)

‘Whether or not he is autistic, I am free to take him wherever I want for socializing on occasion, I took him to McDonald’s and Bela Mall. I make each of my children feel extra special by going to each of their birthday celebrations.’ (P10, Widow, 46 years old, Waterberg)

#### Religious coping

The study found that most of the participants cope by believing and finding comfort in God and through praying to God. Most of the caregivers even if they are not strongly religious have adopted this method in coping with the ASD situation.

Most of the caregivers, who are also mothers of children with ASD, explained how religion assisted them to cope:

‘You also must accept what God has given you because that is the first step to coping. What made me cope was praying telling God all my problems day and night. My friend is a bible I do not run to someone for help or solutions I just kneel and pray. My knees will bring me solutions.’ (P2, Single, 27 years old, Capricorn)

‘Whenever I face any challenge in my life I just kneel and pray. My knees are my weapons so in any stressful situation I just trust in God.’ (P5, Married, 35 years old, Capricorn)

‘I am a Christian, so I do know that God will not give you something that is not suitable for you. If we had to choose no one would choose something bad, we would all choose the good one. I believe in God so whatever challenge I come across I give it to God. I pray and tell him about my troubles or problems. I also know that God will not give you a problem without a solution.’ (P17, Single, 44 years old, Sekhukhune)

### Theme 3: Problem-focused strategies

The caregivers were also found to be utilising the problem-focused coping strategies to handle the stressful situation by tackling the problem that causes the stressful situation. The problem-focused coping strategies include peer group support, professional support, social emotional support, adaptive coping and active coping.

#### Adaptive coping or skills

The study found that parents cope by learning how to live better with their children with special needs. The participants expressed various adaptive skills that they use to cope with their children such as learning sign language to communicate with their ASD children with impaired speech, continuous monitoring, supervision, learning about their behavioural problems and how to manage them, being patient, giving the child attention and teaching the children certain skills as evidenced from the following quotations:

When she hugs me, I always make sure to pay attention to her so I can figure out what she wants. To improve our communication, I’m also attempting to learn sign language.’ (P16, Single, 49 years old, Sekhukhune)

‘I saw his behaviour and knew that if I got into a confrontation with him, he would leave the house and not come back. He won’t have somewhere to go if I pursue him from my house’ (P18, Single, 31 years old, Vhembe)

He needs to be watched closely since he will leave everything behind if you give him a task and walk away.’ (P4, Married, 65 years old, Capricorn)

‘At home, we can relate to him when he needs to use the restroom because there are certain words that he uses that a stranger would not understand. We also take care not to overfeed him dairy products because we know that would cause issues for him. Since my son eats a lot – he may even eat all day – we make sure to restrict items like milk and mayonnaise.’ (P7, Single, 30 years old, Capricon)

‘When I’m at home, I try to teach him how to read and write. He is slow, so I simply make sure I’m patient enough with him.’ (P9, Married, 48 years old, Waterberg)

#### Active coping

It has been discovered that caregivers of children with ASD cope by speaking out for their children and addressing the issue at hand. The following are examples of active coping strategies highlighted by the participants: parent–teacher conferences to discuss potty training, adjusting the surroundings to match the needs of the child and asking for help from neighbours and community leaders to address concerns of rejection of the child. It is evident from the following quotations:

‘“I even took my grievances to the local leaders, and they called a community meeting to let the parents know that their kids shouldn’t be making fun of her at home. Because of his speech impediment, he used to even pass stools on himself in the classroom because his teachers couldn’t understand him. But now that I’ve had to sit down and explain it to his class teacher, I believe the issue is resolved” When he first started coming to this school, I was contacted, and we went to sit down with the instructor to go over the language he uses to ask to use the restroom.’ (P19, Single, 32, Vhembe)

‘There was a time I was called at school when he first attended school here and they and we sat down with the teacher to explain to her the words he uses when he wants to go to the toilet.’ (P20, Married, 36, Vhembe)

#### Peer-group emotional support

The caregivers highlighted that sharing problems with people with the same problems is important to assist each other with the solutions. Nonetheless, every caregiver noted that there are no support groups available for caregivers of children with ASD at special schools or in their local communities. The caregivers stressed how crucial it is for special schools or communities to establish support groups where parents may gather to share their experiences to cope successfully with raising a child with ASD.

It is evident from the following quotations:

‘Another important thing is to have parents’ support groups according to their children’s ASD so that we can share their problems and coping strategies.’ (P20, Married, 36 years old, Vhembe)

‘At this school, there is no support group for parents of children with ASD, ever since my child came here, we have never had a meeting as parents where we share our problems, challenges, and our coping strategies with each other according to our children’s ASD. I tried mentioning it and I ended up fighting with the teachers here because of that.’ (P21, Married, 54 years old, Vhembe)

‘I think a quarterly support group meeting for parents of children with ASD.’ (P23, Married, 53 years old, Vhembe)

#### Professional emotional support

Every caregiver emphasised that after learning of their children’s diagnosis, they had not been advised to seek professional counselling. It was discovered that most caregivers were using alternate coping mechanisms in the absence of professional counselling. Even though they have never been referred, some stressed how much they would like to talk to the psychologist. Very few caregivers of children with ASD reported having gotten counselling from nurses or school teachers, which assisted them a lot with coping:

‘Another teacher at this school helped me a lot to understand my child because it was hard, and she told me that we don’t get to choose which children we want, and God won’t give you a challenge that he knows you won’t be able to deal with. She also told me that God had a purpose by giving her a child like her and a psychologist also helped.’ (P17, Single, 44 years old, Sekhukhune)

‘Another professional nurse tried to console me and said she will be a human being. I went for counselling three times until a time came and I decided to take my child home, but I knew that she will not be a child-like other.’ (P12, Single, 40 years old, Waterberg)

‘The psychologist should be available because it will help other parents who can’t cope well.’ (P15, Single, 27 years old, Sekhukhune)

#### Social emotional support

The study found that caregivers also coped emotionally by getting support from family, friends, spouse and the community at large. Most participants emphasised how supportive their mothers and spouses are at all times, and how this helps them manage the stress and caregiving responsibilities that come with raising a child with ASD. Some said that the other children always help and support them with their caregiving responsibilities, which makes them feel so supported at home. Some emphasised how accepting their neighbours and communities have been of their ASD children. As their families, spouses and neighbours are always there for them, they stressed that it is simpler for them to share their problems with them.

It is evident from the following quotations:

‘I cope because, I stay with my husband and children, they support me, they don’t have a problem.’ (P8, Single, 29 years old, Capricorn)

‘In the community where we stay, I do not see a problem they do accept her. To be honest my mom is my support system, she helped me a lot with my child when I could not accept that my child is autistic. Whenever am stressed with anything she is the first person I talk to.’ (P14, Single, 43 years old, Waterberg)

‘We sat down as a family and her father is someone who loves his children a lot and he was so supportive. What made me cope was the family support they supported us a lot.’ (P11, Single, 25 years old, Waterberg)

‘The support that we provide to each other as the family keeps me going. My other children help take care of the child with ASD and they understood her condition.’ (P19, Single, 32 years old, Vhembe)

‘My brother is the one person I can talk to when I have problems, he helps me come up with solutions and he gives me advice regarding any situation I would be facing at that time, my brother is my support system.’ (P26, Married, 43 years old, Vhembe)

## Discussion

This research explored and described the coping strategies adopted by the caregivers of children with ASD in the Limpopo province, South Africa. The study found that caregivers of children with ASD perceived raising or having a child with ASD as a threat, challenge and loss, and this generated emotions. They describe their situation as a stressful one because there are limited integrated social support services focusing on caregivers or families of children with ASD. As a result, the caregivers employed various strategies that have positive and negative effects to overcome their stressful situation. The strategies include the positive and negative emotion-focused strategies as well as the problem-focused strategies. The negative coping mechanisms include denial, release of emotions, isolation of self and the child, overprotection, cognitive distortion, punishment and negative self-talk, whereas the positive coping mechanisms include reframing, acceptance, positive appraisals, religious and self-talk. The problem-focused coping strategies include adaptive coping, active coping and acquiring professional, social and peer emotional support.

In this study, the negative emotion-focused coping techniques negatively influence the caregivers’ ability to adjust to the situation. The emotional distress of the caregivers was not lessened by using these negative coping mechanisms. Despite having raised the child for many years, several caregivers were still becoming emotional when describing their circumstances. This is in line with the previous studies that questioned the effectiveness of emotion-focused strategies in parents of children with ASD (Side & Kumar 2022; Miranda, et al. 2019; Zhou et al. 2019). Similarly, emotion coping strategies such as denial are reported to have a negative impact on caregivers’ stress (Amireh 2019; Bozkurt, Uysal & Düzkaya 2019). ASD children are deprived of the opportunity to explore the outside world because of negative coping mechanisms adopted by their caregivers such as overprotection, punishment and isolation. These strategies may lower the self-esteem of the child which can result in a lifetime of underachievement and failure to reach their full potential (Berjot & Gillet 2011; Ishtiaq et al. 2020; Obbarius et al. 2021). The study’s setting and the participants’ socioeconomic background may have had a role in their adoption of these strategies. Most of the participants came from deeply rural areas, were less educated and have less access to or understanding of psychotherapy.

However, other caregivers maintained hope and optimism by using the positive emotion-focused coping mechanisms, which reduced their emotional distress (Ang & Loh 2019). The participants with dual roles of being a mother and a teacher of children with ASD adopted these strategies. Caregivers who were using the positive emotion- and problem-focused coping techniques were found to have positive stress. It is possible that their in-depth understanding and passion for ASD led to the adoption of these strategies. All the caregivers emphasised that although it was initially difficult, however, accepting the child was the most important and crucial step in learning to deal with the situation effectively. Similarly, the previous studies reported acceptance as one of the more effective mechanisms in coping with stress (Brown et al. 2020; Balubaid & Sahab 2017), and this is associated with increased life satisfaction (Havighurst et al. 2020). Only after awareness and acceptance that a child has ASD, the caregiver would proceed to the next stage of training and skill development (Pisula & Banasiak 2020).

The belief in or support of religion was another strategy that was frequently cited as providing caregivers with hope and courage to deal with the situation like the previous studies (Craig et al. 2020; Davis III & Kiang 2020; Furrukh & Anjum 2020). This is in line with the previous research religious coping was seen to be more commonly employed as compared to other coping styles (Selvakumar & Panicker 2020; Side & Kumar 2022; Wei-Chih et al. 2023). The religious practice was reported to give them peace of mind and helped them to endure the caregiving situation (Loewenstein et al. 2019). The caregivers who were using a coping mechanism such as reframing spoke openly about their circumstances with confidence and showed no evidence of emotional strain. Similarly, the use of positive reframing and positive reinterpretation assisted in overcoming and adapting to the current difficult situation (Demšar & Bakracevic 2023; Loewenstein et al. 2019; Selvakumar & Panicker 2020). In this study, the caregivers adopted positive strategies to convince themselves about the actual conditions of raising a child with ASD so they could see the situation as non-threatening rather than threatening.

## Recommendations

According to the study, caregivers of children with ASD in Limpopo province are not given enough support to help them deal with the stress of raising an ASD child. However, the National Mental Health Policy Framework and Strategic Plan 2013–2020 (Department of Health 2012), highlighted that maximum support should be provided to families and caregivers of those with mental illness to broaden the network of support and care. A ministerial task team, therefore, should be established to oversee the policy’s implementation. Integrated services and social support for families of children with ASD are the most crucial elements that need to be closely observed in a rural area like Limpopo province. Health care facilities and special schools shall be monitored and assessed by the Department of Health, basic education and social development at the district level for their ability to provide integrated services and social support to families and caregivers of individuals with ASD. Semi-structured interviews and situation analysis with the key stakeholders, such as parents, caregivers, families, religious leaders, nurses, social workers, psychologists and special education teachers, might be used to accomplish these. Furthermore, to provide caregivers with the most social support possible, coordination and collaboration across various community resources such as social services, health and religious groups are required.

## Conclusion

This study utilised Lazarus and Folkman’s Transactional Model of Stress and Coping theoretical framework and qualitative methods to determine and characterise the distinct coping strategies employed by caregivers of children with ASD. This study showed that most caregivers have been able to cope, adjust and often grow up to fulfil the demands of these children, even though there is a peek at the everyday stress they suffer. In conclusion, the coping methods that have been identified can be integrated into intervention programmes and serve as a guide for specialised institutions that offer more extensive knowledge and assistance to families who are caring for children with ASD. This study will assist mental health care providers in giving caregivers greater care and support through counselling and education on coping mechanisms and adaptation techniques.
